# Stimulatory Effect of Mammalian Exosomes on Intradermal Fibroblast Proliferation

**DOI:** 10.1111/jocd.70027

**Published:** 2025-02-07

**Authors:** Alina Kilasoniya, Lidia Zabegina, Andrey Shalaev, Anna Artemyeva, Anastasia Malek

**Affiliations:** ^1^ Campus de Los Jerónimos Universidad Católica de Murcia Murcia Spain; ^2^ Subcellular Technology Laboratory, Department of Hematology and Chemotherapy and Department of Radionuclide Diagnostics N.N. Petrov National Medical Research Center of Oncology St. Petersburg Russia

**Keywords:** exosomes 1, extracellular vesicles 2, fibroblast 3, skin 4

## Abstract

**Background:**

This study investigates the potential of extracellular vesicles (EVs) in skin regeneration and rejuvenation. EVs, nanoscale vesicles released by various cell types, play a crucial role in intercellular communication.

**Objective:**

To reaffirm the pivotal role of blood‐derived exosomes in intercellular communication and their potential for skin tissue regeneration, leveraging existing research, including human data, to advocate for exosomes as a viable cell‐free therapy for skin health.

**Methods:**

The study employs a novel isolation technique combining PEG and Dextran with ultracentrifugation to extract EVs from plasma. Characterization techniques, including NTA, AFM, Cryo‐TEM, and FC, confirm the successful isolation and characterization of EVs.

**Results:**

The study demonstrates positive effects of blood‐derived EVs on fibroblast proliferation, collagen, and elastin production in murine and human models. Despite advancements, challenges persist in obtaining consistent EVs quality and concentration. The findings support the clinical relevance of EVs in skin health and suggest potential applications for skin rejuvenation. Future research directions and study limitations are also discussed, contributing to the evolving understanding of EVs‐based therapies.

## 1 Introduction

Extracellular vesicles (EVs), including exosomes, are released by various cell types and contain a range of cellular products such as RNA, DNA, proteins, lipids, amino acids, and metabolites [1]. EVs participate in intercellular communication and are of nanoscale size, resembling bubble‐like membranous structures. These vesicles are found in different plants [2] and various organic fluids, such as blood, urine, saliva, breast milk, and cerebrospinal fluid. There are different categories of EVs, including apoptotic bodies (500–1000 nm, released during cell apoptosis), microvesicles (100–, nm, resulting from plasma membrane evagination), and exosomes (50–200 nm) [3]. Exosomes are a specific subtype of EVs formed through the fusion of multivesicular bodies with the plasma membrane. They are distinct from other EVs due to their smaller size and the presence of specific exosomal markers like CD9, CD63, CD81, and others [4].

Extracellular vesicles, particularly exosomes, play a vital role in cell‐to‐cell signaling and have potential applications as biomarkers for disease diagnosis, prognosis, therapy, and tissue regeneration. In the context of skin health, exosome‐mediated cell‐to‐cell communication is essential for maintaining tissue balance and cellular function, facilitating wound healing through proangiogenic, anti‐inflammatory, and antifibrotic properties. As a result, exosomes derived from blood have been recently introduced into the field of skin rejuvenation for their anti‐aging properties [5].

Exosomes, extracellular vesicles derived from stem cells, are emerging in cosmetic dermatology for their ability to influence cellular processes like collagen production and rejuvenation. Recent studies show they can reverse skin aging, improve elasticity, and enhance wound healing. Their potential positions exosomes as key tools for noninvasive regenerative therapies in aesthetics [6].

Exosomes have been demonstrated to enhance the migration of cells and stimulate collagen synthesis in human dermal fibroblasts (HDFs). In the same context, these blood‐derived exosomes penetrate the skin layer and significantly boost the production of collagen and elastin, two key components for skin rejuvenation. This has led to a growing demand for simple, efficient, and cost‐effective exosome isolation techniques. Currently, obtaining exosomes with consistent quality and high concentration remains a significant challenge [7].

Recent research highlights the potential of exosomes in regenerative dermatology. Sheykhhasan et al. [1] discuss their dual roles in neurodegenerative diseases, while Salazar‐Bermeo et al. [2] explored the plant‐derived exosomes from grapefruit and tomato for targeted skin applications. Vu et al. [8] show that exosomes from bone marrow mesenchymal stem cells enhance fibroblast migration and collagen production, and Chen et al. [9] examine their effects on wound healing in animal models, indicating promising clinical applications for skin regeneration and injury treatment. In this Journal, Olumesi and Goldberg [10] provide an overview of exosomes in aesthetic dermatology, reviewing their efficacy in skin rejuvenation, wound healing, and scar treatment, and highlighting the necessity of more extensive studies to establish safety and long‐term benefits.

Several approaches for exosome isolation have been developed, including differential ultracentrifugation, size‐based ultrafiltration, and microfluidics‐based platforms. Specific exosome precipitation methods, such as immunoaffinity capture‐based techniques or lectin‐based purification, and non‐specific precipitation using polyethylene glycol (PEG), alginic acid, and hydrophobic binding, are also available. According to the International Society for Extracellular Vesicles (ISEV) [11] guidelines, isolated EVs are classified as exosomes if they fall within the size range of 30–200 nm, possess a characteristic spherical shape, feature a bilayer membrane, and are enriched with exosomal markers [12].

To enhance exosome isolation, future efforts should utilize advanced technologies like microfluidics and AI‐assisted separation, develop affinity‐based materials for improved purity, and standardize protocols for consistency. Optimizing two‐phase polymer systems and using hybrid techniques that combine ultracentrifugation with chromatography could also increase efficiency. These strategies aim to improve the quality and reliability of exosome research and applications [13].

In this study, PEG and Dextran [14] was used in combination with differential ultracentrifugation to isolate exosomes from plasma [3]. These exosomes, obtained from the blood of mice, were subsequently administered through mesotherapy to evaluate their impact on skin fibroblasts, collagen, and elastin levels [15] over a period of 3–10 days. A similar experiment was conducted on a healthy volunteer for 7 days to ascertain the compatibility of the results observed in animals with those in humans.

## 2 Materials and Methods

### 2.1 Isolation and Purification of Exosomes

First step corresponds to the isolation of exosomes using the two‐phase polymeric system method.

For blood preparation, blood was collected intravenously with ethylenediaminetetraacetic acid (EDTA). To separate the plasma, it was centrifuged for 15 min at 1500 ×*g* at 4°C.

For plasma preparation, the plasma was centrifuged for 10 min at 300 ×*g*, 1500 ×*g*, and 2500 ×*g*, collecting the supernatant and filtering it through a 0.22‐μm pore diameter filter.

To isolate exosomes from a plasma sample, it is necessary to prepare two heavy polymer solutions, one for plasma and one for the washing solution. One solution consists of 80.7 μg of dextran (Dextran 450–650 kDa) and 21.37 μg of PEG (Polygle 20 kDa). One solution was added to the plasma and the other to phosphate‐buffered saline (PBS) (for the washing solution).

For the washing solution, 150 μL of dextran and 50 μL of PEG were added to PBS.

This mixture was incubated for 60 min on a multi‐vortex at 4°C, stirring every 20 min, then centrifuged for 10 min at 1000 ×*g*. We removed the upper phase (approximately 1400 μg) from the Eppendorf plasma and replaced it with the upper phase of the washing solution.

After centrifuging for another 10 min at 1000 ×*g*, we removed the upper phase. In the lower phase, we resuspended the polymer pellet and exosomes at 100 ×*g*. We then unspun it for 3 min at 6000 ×*g* in a microspin and transferred the supernatant containing exosomes to a separate tube.

### 2.2 Nanoparticle Tracking Analysis (NTA)

The size of exosomes and their concentration in suspensions were determined by NTA using the NanoSight NS300 (Malvern Instruments, UK) analyzer, equipped with a blue laser (45 mW at 488 nm) and a Scientific CMOS camera (Hamamatsu Photonics K.K., Japan). Recording and data analysis were performed using the NTA software 3.4. The measurements were carried out at camera level: 11, shutter slider: 890, slider gain: 125, and threshold level: 5. The following parameters were evaluated during the analysis of recordings monitored for 30 s: the average hydrodynamic diameter, the mode of distribution, the standard deviation, and the concentration of vesicles in the suspension.

Exosomes were isolated from 5 mL of mice and 15 mL of human blood by using the two‐phase polymeric system method to the purification protocol described earlier with some modifications. In order to characterize mouse and human exosomes, their size distribution and concentration were measured by NTA. In the purified samples of blood exosomes, the following parameters were measured: size mode and concentration of vesicles in suspension.

### 2.3 Atomic Force Microscopy (AFM)

The morphology of exosomes were determined by AFM using the NT‐MDT Solver Bio scanning probe microscope (Molecular Devices and Tools for Nanotechnology, Moscow, Russia) in the tapping mode with probe NSG01_DLS. Results were processed by the following programs: Image Analysis (NT‐MDT, Russia) and Gwyddion 2.56 (gwyddion.net).

### 2.4 Transmission Cryo‐Electron Microscopy (Cryo‐TEM)

Visualization was performed by cryo‐TEM on Jeol JEM‐2100 microscope at the Research Resource Center for Molecular and Cell Technologies of Saint Petersburg State University.

### 2.5 Flow Cytometry (FC)

To determine the presence of exosomal surface markers, bead‐assisted flow cytometry was used. Exosomes were nonspecifically absorbed on the surface of aldehyde‐sulfate latex beads (A37304, Invitrogen, USA). Tetraspanins CD9, CD63, and CD81 were detected using antibodies conjugated with fluorescent labels FITC (CD9‐FITC, ab18241, Abcam, Cambridge, UK), PE (CD63‐PE, 353004, BioLegends, San Diego, CA, USA) and PerCP (CD81‐PerCP, 349 508, BioLegends, San Diego, CA, USA). Cytoflex analyzer and CytExpert software (both from Beckman Coulter, Brea, CA, USA) were used for sample measurement and for following data processing, consequently.

### 2.6 Cell Culture

Adult human skin fibroblasts primary cell culture was established as described [16] with slight modification. Cells were cultured in DMEM/F12 medium containing 2 mM L‐glutamine and 10% fetal calf serum. Fibroblasts of third passage were seeded on a 6‐well plate at a density of 8 × 10^6^ cells/cm^2^ and placed in standard conditions of CO_2_ incubator. After 24 hours of incubation, standard media was replaced by FCS‐free medium, and human plasma exosomes were added up to a final concentration equivalent to the estimated concentration of exosomes in plasma (10^13^ per mL). Control cells were incubated in FCS‐free medium. Detailed experimental conditions were described previously [17]. Imaging was performed following 24 h of cultivation by phase contrast microscope “MIB‐R” supplied with a digital camera (LOMO, Russia) using ×30 and ×150 objective lenses. Culture media and cells were harvested following 48 h of cultivation for further analysis.

### 2.7 ELISA

The concentration of collagen type I, elastin, and soluble CD44v (v6) in culture media was determined by enzyme immunoassay (ELISA) using Abcam (USA) test systems (ab285250, ab239433, ab45912) according to the manufacturer's method. Measurement was performed with the Varioskan LUX Multimode Microplate Reader (Thermo Scientific, USA) in five replicates.

### 2.8 RNA Isolation and RT‐PCR

RNA was extracted from cell pellets using RNA isolation Kit (BioSilica, Russia) following the manufacturer's protocol based on conventional column‐spin technology. RNA concentration and purity were confirmed by spectrophotometric ratios using absorbance measurements at wavelengths of 260/280 nm with a NanoDrop 2000 (Thermo Scientific, USA). For mRNA quantification, cDNA was synthesized using reverse transcriptase M‐MuLV–RH, and quantitative PCR was performed with HS‐Taq DNA polymerase (both from BiolabMix, Novosibirsk, Russia) in accordance with the manufacturer's protocols. Primers for PCR (COL1A1‐FW: CCAGCCACAAAGAGTCTACA; COL1A1‐RV: TCTGTACGCAGGTGATTGGT; ELN‐FW: GGCAAACCTCTTAAGCCAGT; ELN‐RV: AGACACTCCTAAGCCACCAA; CD44‐FW: TGCCGCTTTGCAGGTGTATT; CD44‐RV: CCGATGCTCAGAGCTTTCTCC; GAPDH‐FW: GAAGGTCGGAGTCAACGGAT; GAPDH‐RV: GCCATGGGTGGAATCATATTG) were synthesized by “Lumiprobe” Ltd. (Saint‐Petersburg, Russia). Assays were performed using the CFX96 Touch System (BioRad, USA) in triplicate; specific mRNA expression data were normalized versus GAPDH. Results of two independent experiments were averaged, and statistical significance was estimated by Mann–Whitney test.

### 2.9 Intradermal Application

Were selected four 73‐day‐old mice and administered 60 μL of exosomes and 60 μL of PBS to them through intradermal mesotherapy, resulting in the formation of papules on the postero‐inferior dorsal area. As a control, saline solution was used in the contralateral area. After 4 and 11 days, we performed punch biopsies (2 mm diameter) on both regions of two mice each day to obtain biomaterial for histological analysis.

For the chosen healthy subject, 100 μL of exosomes and 100 μL of PBS were administered using intradermal mesotherapy in the right and left lateral neck areas, respectively. As a control, saline solution was used in the contralateral area. After 7 days, punch biopsies (2 mm diameter) were conducted on both regions under local anesthesia to obtain biomaterial for histological study.

### 2.10 Histological Analysis

Samples were fixed in 10% buffered formalin, dehydrated in increasing concentrations of ethanol (70%, 90%, and 100%, each for 30 min), immersed in xylene for 30 min twice, and in liquid paraffin for 30 min once for impregnation. These specimens were then embedded in paraffin and sectioned at 5 μm in thickness. Hematoxylin and eosin stain, silver staining to evaluate elastin fibers, and Masson's trichrome staining were carried out in order to evaluate collagen fibers.

### 2.11 Consistency Challenges

To ensure consistent EV quality and concentration, we have implemented strict quality control measures, including a novel PEG and dextran isolation method with optimized ultracentrifugation. We are standardizing procedures, optimizing centrifugation parameters, and using uniform polymer solutions. Each step is documented and reviewed to reduce variability, and we are exploring scalable techniques for larger studies. These efforts aim to enhance the reliability and reproducibility of EV research outcomes.

## 3 Results

Aqueous solutions of 25–45 kDa PEG and 450–650 kDa DEX form a two‐phase that can be used to isolate EVs from biological fluids. The system was reported using human plasma as the solvent; however, the formation of two phases was not observed. Apparently, the complex composition of plasma interfered with the polymer solution and affected the conditions required for phase separation.

### 3.1 Characterization of Isolated Exosomes

#### 3.1.1 NTA

Parameters referring to the size and concentration of EVs in suspension were measured in the blood samples.

**FIGURE 1:**
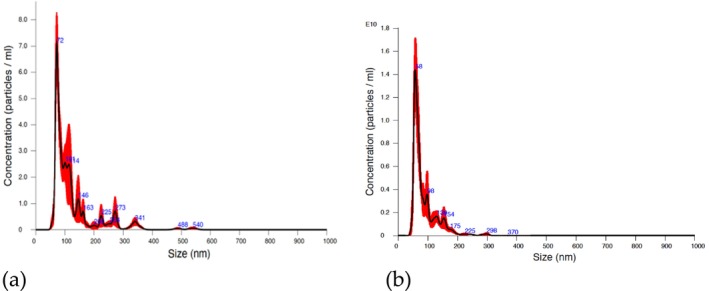
Characterization of blood EVs from mouse (a) and human (b). Histogram of particle concentration and size distribution of blood EVs from mouse measured by nanoparticle tracking analysis (NTA).

These parameters were determined as follows: mice mode 73.5 ± 2.3 nm and concentration 2.85e+11 ± 3.59e+10 particles/ml and human mode 58.1 ± 1.7 nm and concentration 4.75e+11 ± 4.49e+10 particles/ml (Figure 1).

#### 3.1.2 AFM

**FIGURE 2:**
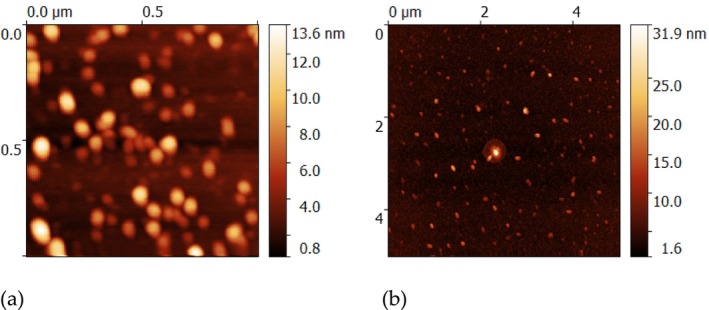
Characterization of EVs size and morphology by atomic force microscopy (AFM). (a) Mouse. (b) Human.

AFM estimated the surface topology of EVs from blood samples, observing individual spherical to oval‐shaped particles corresponding to vesicular topology with diameters ranging between 40 and 90 nm and heights between 20 and 40 nm. In addition, several small particles with heights of approximately 13 nm were also observed (Figure 2).

#### 3.1.3 Cryo‐TEM

**FIGURE 3:**
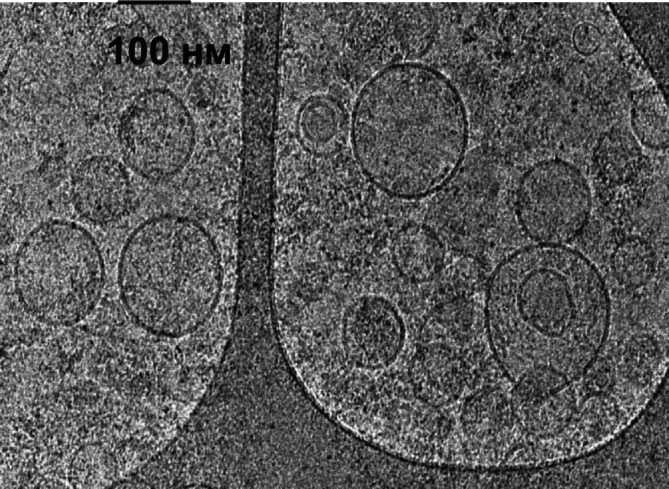
Cryo‐EM images of human EVs.

The structure and morphology of EVs were characterized by Cryo‐EM (Figure 3). Cryo‐EM images revealed the spherical shape of EVs. The average size of EVs ranged from 28 to 205 nm in diameter. A small number of larger vesicles (up to 200 nm) were also observed. Cryo‐EM images confirmed the presence of clearly discernible lipid bilayer structures.

#### 3.1.4 Fc

**FIGURE 4:**
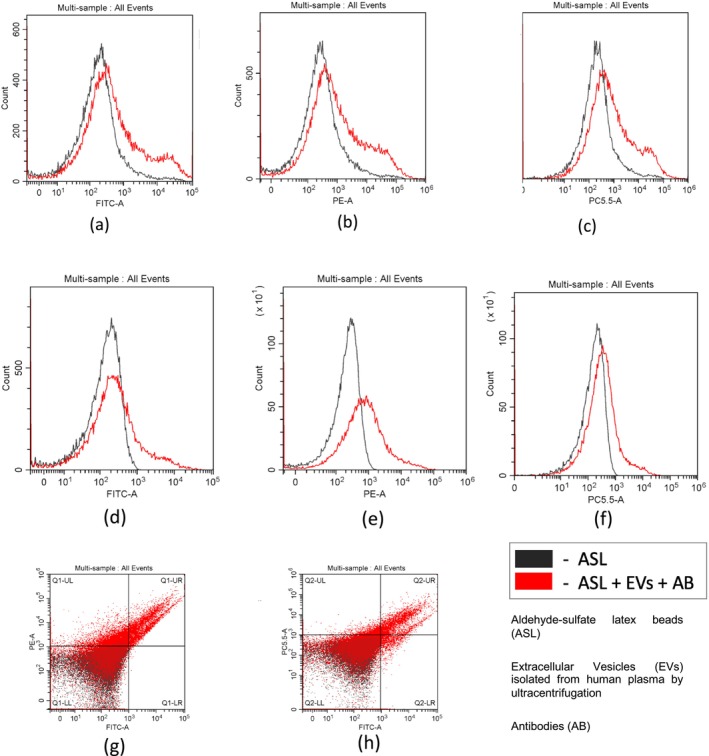
Flow cytometry analysis of EVs isolated from mouse (a–c) and human (d–h) plasma. Latex beads were incubated with EVs, followed by incubation with antibodies against CD9 (a, d); CD63 (b, e) and CD81 (c, f). Beads incubated with AB only were used as a negative control in all experiments (black line). Combined expression of tetraspanins CD9/CD63 and CD9/CD81 on mouse EVs is shown as well (g and h, correspondently).

Semiquantitative flow cytometry analysis was used to evaluate expression of several tetraspanins (CD9, CD63, and CD81) typically presented on the exosomal surface (Figure 4). In these experiments, antibodies generated against human proteins were used. As human proteins share amino acid sequence identity with mouse proteins (CD9: 77%, CD63: 67%, CD81: 93%), we expected to see a positive signal with mouse EVs. As shown in Figure 4, both human and mouse vesicles were positive for exosomal markers. Moreover, the combined expression of CD9/CD63 and CD9/CD81 on a significant part of mouse vesicles was estimated by two‐channel plotting (Figure 4g,h).

### 3.2 Effect of Blood Exosomes on Activation of Adult Human Skin Fibroblasts

**FIGURE 5:**
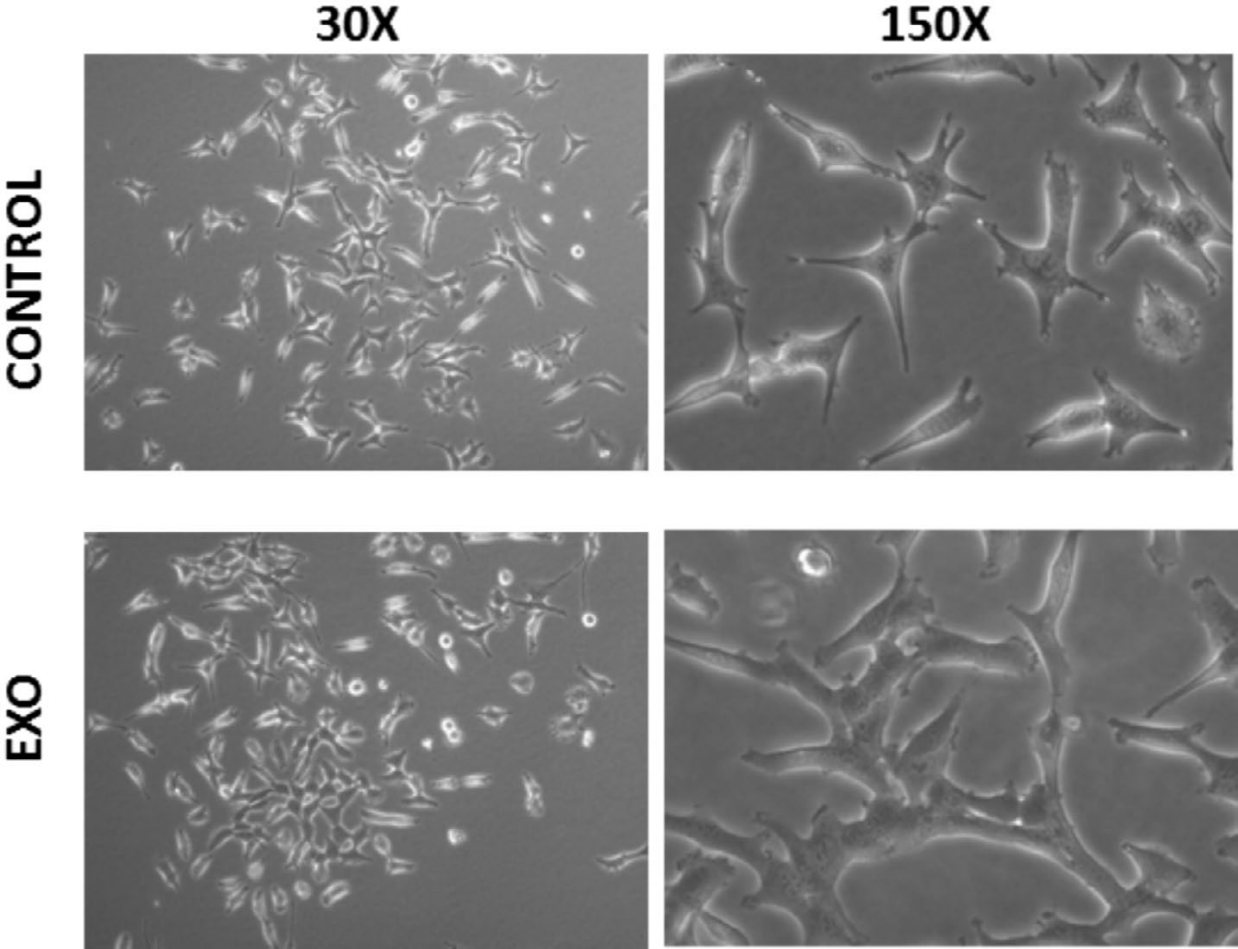
Adult human skin fibroblasts cultured in standard conditions (upper panel) and under exposition to plasma exosomes (10^13^ per mL) during 24 h.

**FIGURE 6:**
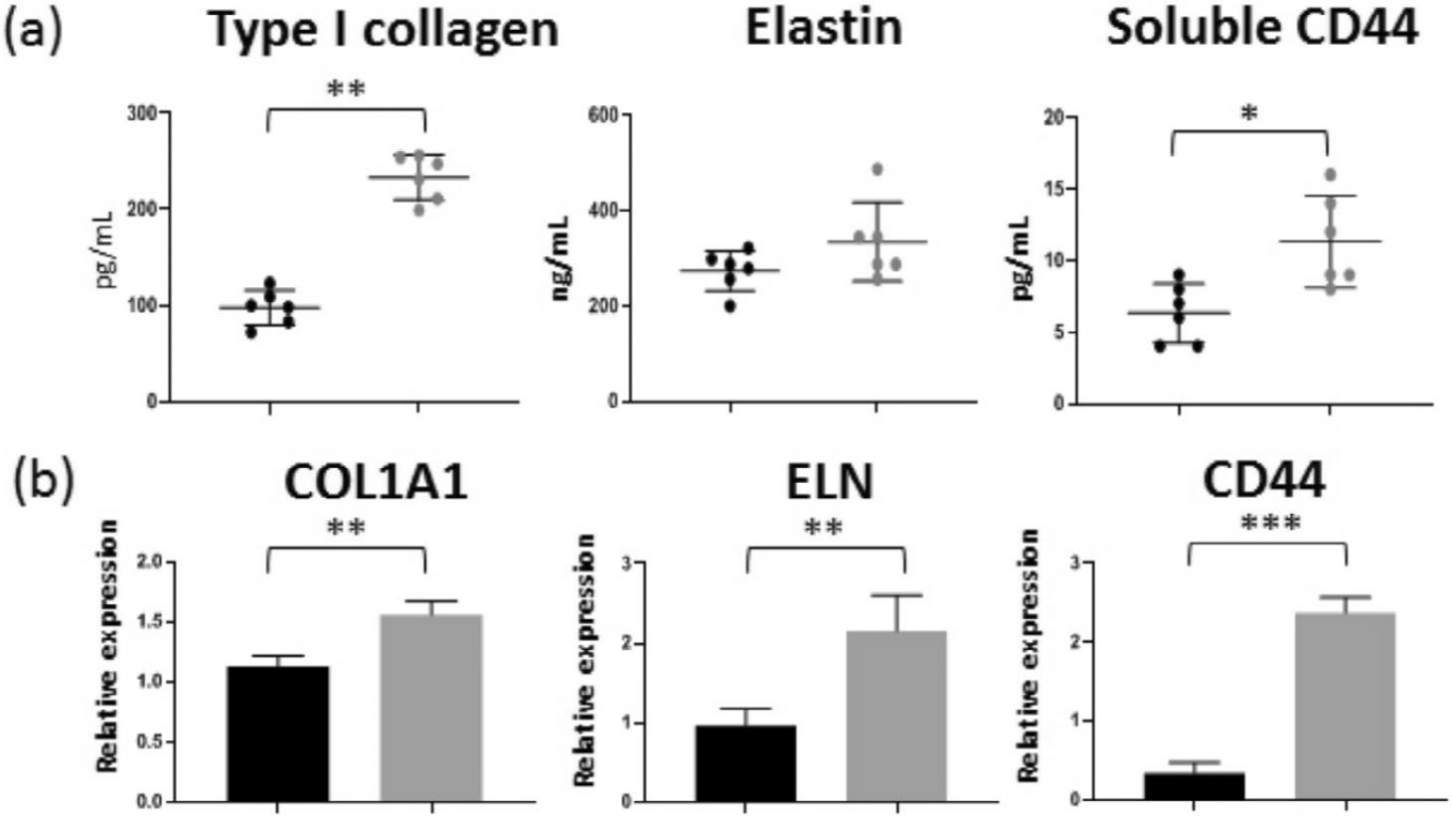
Effect of incubation of human skin fibroblasts with plasma exosomes during 48 h. (a) Measurement of ECM components in conditional media by ELISA. (b) Analysis of gene expression by RT‐qPCR. Black color corresponds to standard conditions of culturing; gray color corresponds to experimental conditions. Statistical significance of the observed difference was estimated by the Mann–Whitney test (***p < 0.0005; **p < 0.005, *p < 0.05).

Plasma exosomes were applied to primary cultures of skin fibroblasts in concentrations equivalent to their estimated concentrations in plasma. Alteration of cell morphology was observed after 24 h of incubation: cells formed multiple sites of intercellular contact and adhered to each other (Figure 5). The concentrations of selected ECM (collagen I type, elastin, and soluble form of CD44) components were increased in the conditioned media of cells cultured with plasma exosomes over 48 h. Moreover, under experimental conditions, the expression of the corresponding genes (COL1A1, LNE, CD44) was activated, which can be assumed to be the reason for the increased secretion of the ECM components by fibroblasts incubated with exosomes (Figure 6).

### 3.3 Effect of Blood Exosomes on Activation of Fibroblasts, Collagen, and Elastin Deposition in a Skin Rat and Human Model

**FIGURE 7:**
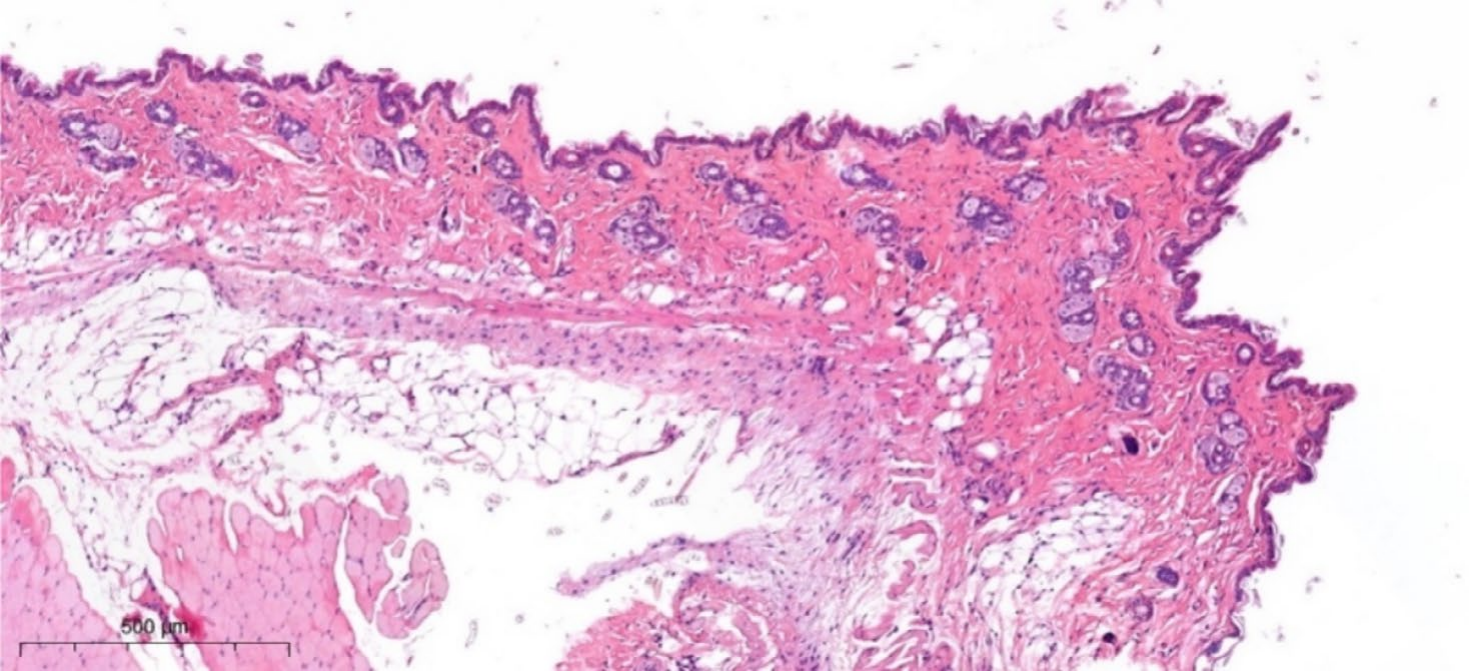
Mouse skin in the EV injection area. Fibroblast proliferation. Hematoxylin and eosin staining.

After subdermal injection of EVs, fibroblast proliferation was detected in the mouse skin after 11 days (arrow) (Figure 7).

**FIGURE 8:**
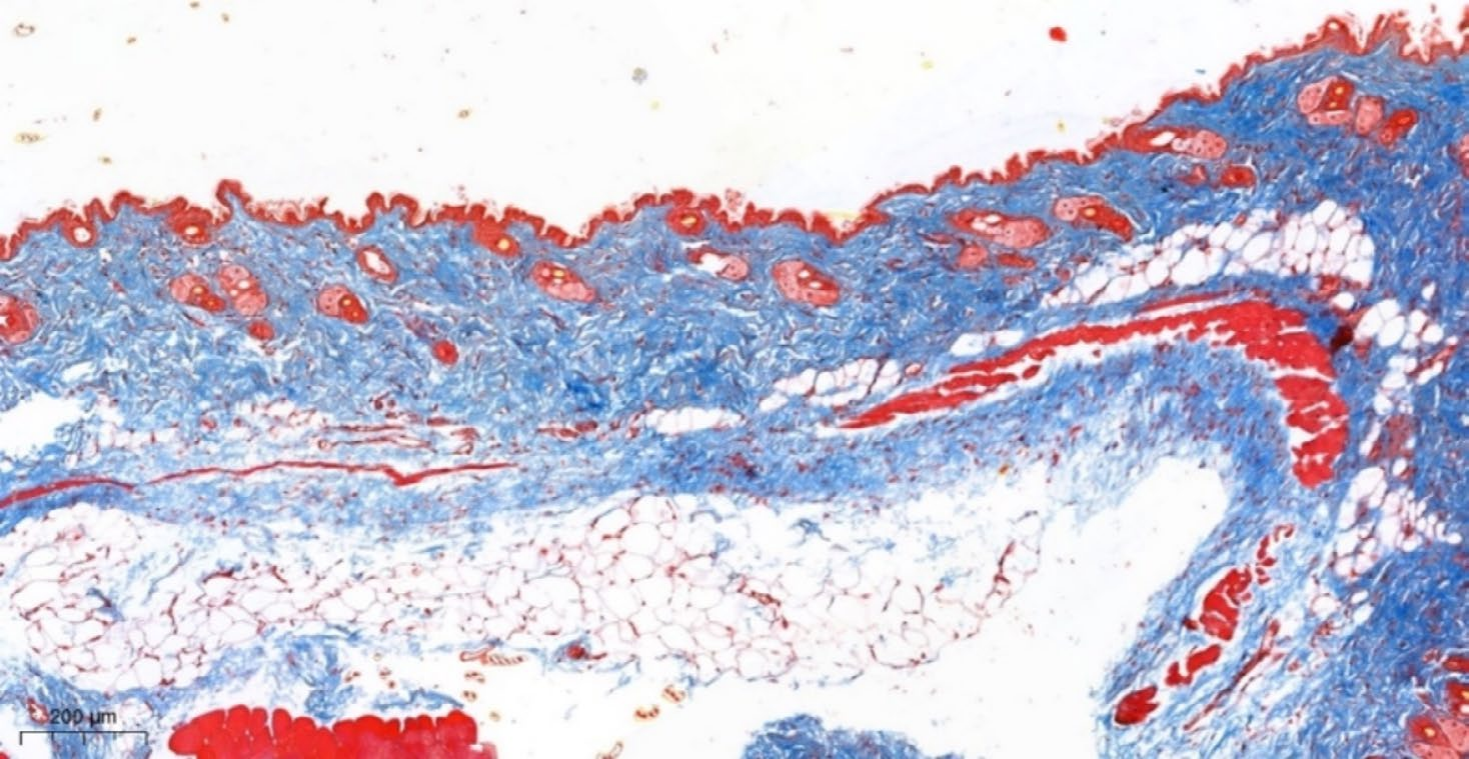
Mouse skin in the EVS injection area. Fibroblast proliferation and production of loose collagen, less basophilic compared to dermal collagen. Masson stain (with aniline blue).

When the sample is stained according to Masson (with aniline blue), in the area of injection and proliferation of fibroblasts, collagen production is detected with a looser, less compact structure, compared to dermal collagen, with a less pronounced basophilia (Figure 8).

**FIGURE 9:**
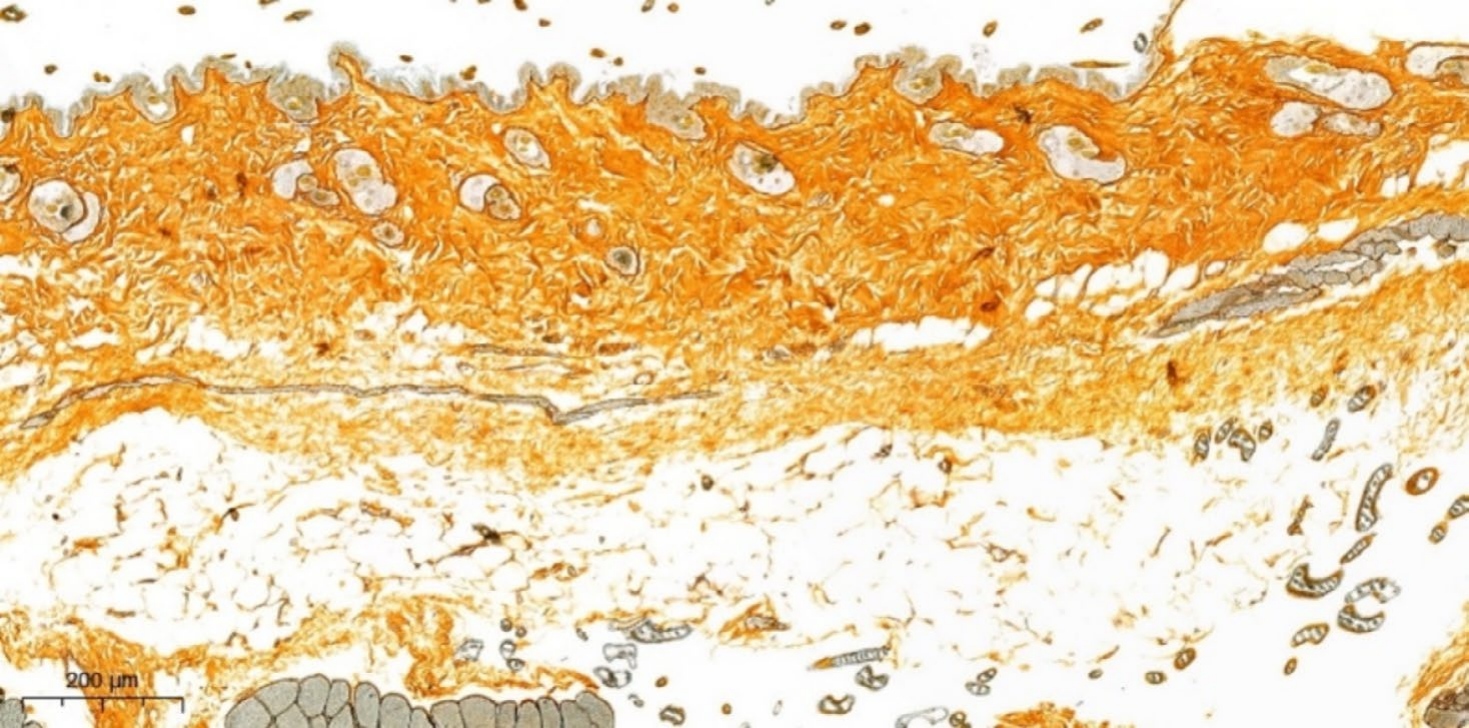
Mouse skin in the EV injection area. No elastosis (destruction) of the reticulin fibers was detected. Silver impregnation stain.

When reticulin fibers are impregnated with silver in mouse skin, the fibers are thin, more pronounced around the structures of skin appendages, muscle bundles, and blood vessels; there is no elastosis. After the injection of EVs, no increase in the severity of elastosis is observed (Figure 9). When staining reticulin fibers by silver impregnation, in comparison with the control sample, no differences (decrease) in the number of reticulin fibers are detected, and there are no signs of their destruction.

**FIGURE 10:**
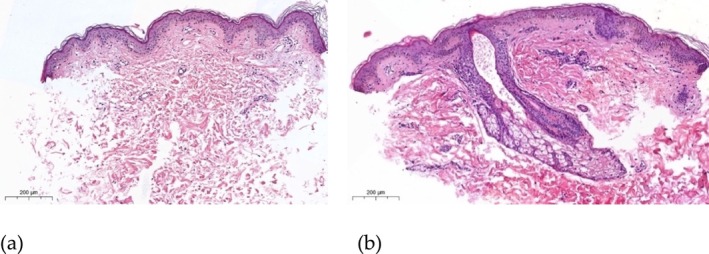
Skin of the upper third of the lateral surface of the neck: (a) control sample (without injection); (b) in the EV injection area. The initial proliferation of fibroblasts is determined in the superficial parts of the papillary dermis and periadnexally. Hematoxylin and eosin staining.

With intradermal injection of EVs into the skin of the lateral surface of the upper third of the neck of a healthy volunteer, an initial proliferation of fibroblasts is observed 7 days after injection, more pronounced subepidermally and around the skin appendage (Figure 10).

**FIGURE 11:**
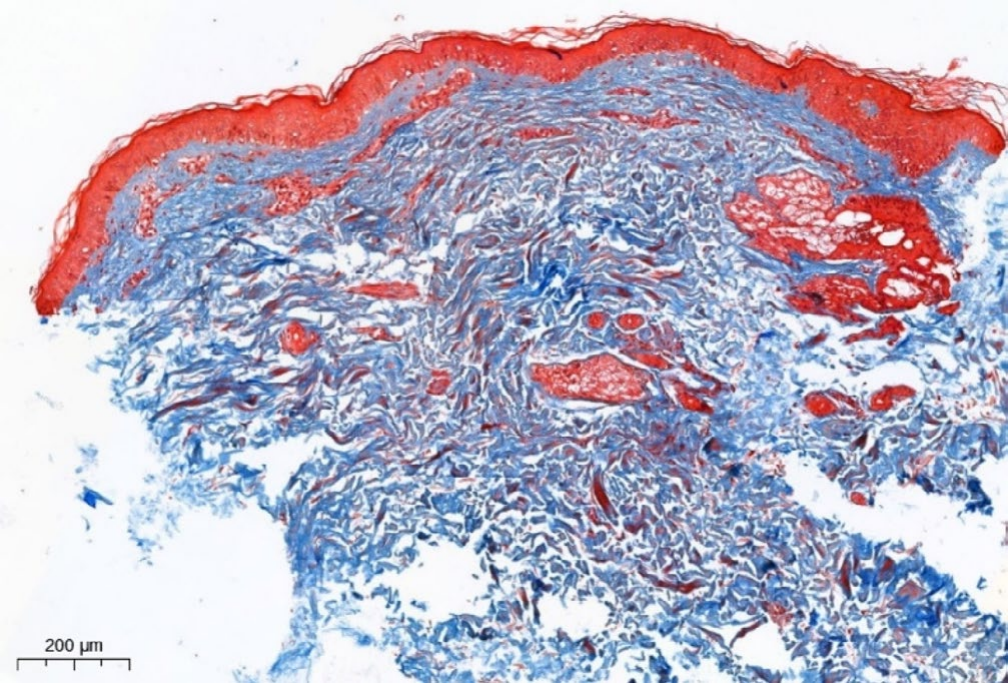
Skin of the upper third of the lateral surface of the neck in the EV injection area. Increased production of loose collagen in the superficial parts of the papillary dermis and perianexally. Masson stain (with aniline blue).

Masson staining (with aniline blue) in the EV injection zone reveals the accumulation of loose collagen in the areas of fibroblast proliferation (Figure 11).

## 4 Discussion

This study reconfirms the vital role of blood‐derived exosomes in intercellular communication and their potential for tissue regeneration, particularly in skin health. Building on previous research, including human data, this study provides strong evidence supporting the use of exosomes as a promising cell‐free therapy for skin regeneration [6].

This study affirms the effective use of heavy polymers like PEG and Dextran, coupled with differential ultracentrifugation, for isolating exosomes from plasma. The success is evident in the thorough characterization of the obtained exosomes. Exosome isolation techniques, crucial for various biological applications, must efficiently separate them from cellular debris. Ultracentrifugation, especially preparative ultracentrifugation, comprises 56% of utilized methods in exosome research. The gold standard [7], differential ultracentrifugation, relies on density and size differences, but there is a growing need for simpler and cost‐effective techniques despite ongoing challenges in achieving consistent exosome quality and concentration [18].

This research employs various techniques, including NTA, AFM, Cryo‐TEM, and FC, to comprehensively characterize exosomes [12, 19]. The detailed analysis of size, morphology, and surface markers contributes to a thorough understanding of exosome nature. Characterization is essential for deciphering their biological interactions. NTA tracks Brownian motion, offering quick measurement of exosome concentration, size distribution, and phenotype with minimal sample preparation. AFM studies exosomes at the nanoscale, providing insights into abundance, morphology, biomechanics, and biomolecular composition under native conditions. Flow Cytometry characterizes exosomal surface proteins, measures size and structure, and identifies cellular origin, with new‐generation instruments enhancing sensitivity for direct detection of smaller particles in suspended exosomes [20].

This study explores the intricate process of skin aging, emphasizing the degradation of key extracellular matrix components like collagen, elastin, and hyaluronic acid. Recent research underscores the regenerative potential of exosomes in counteracting aging effects, showcasing their ability to promote cell migration, proliferation, and blood vessel formation. The study investigates the impact of blood‐derived exosomes on fibroblast activities, revealing positive effects on proliferation, collagen, and elastin production in both murine and human models. These findings suggest the potential of blood exosomes as candidates for protecting against skin aging [21].

This pilot study, with a limited sample size, may affect the reliability and generalizability of its results. While it includes both an in vivo murine model and a human subject, further research, including randomized trials and larger animal models, is essential to validate the efficacy and safety of EV‐based therapies for skin rejuvenation. Future studies will address these needs.

## 5 Commercial Applications

Exosomes are gaining traction in the cosmetics industry for their regenerative and anti‐inflammatory properties, and their ability to boost collagen and elastin synthesis. Their deep skin penetration and activation of internal regenerative mechanisms position them as a transformative ingredient in premium skincare. The industry is developing innovative formulations that utilize exosomes, paving the way for personalized treatments in “precision cosmetics.” Collaboration between academia and cosmetics companies could enhance the commercial use of exosome‐based therapies, appealing to consumers and investors interested in scientifically backed innovation [2, 9].

## 6 Regulatory Aspects

The commercial application of exosomes in cosmetics faces significant regulatory challenges due to their biological origin, which subjects them to stricter scrutiny than traditional ingredients. Regulatory bodies like the FDA and EMA evaluate these products for their potential human impact, but there are no established regulations specifically for exosome‐containing cosmetics [22].

In the U.S., the FDA has warned against unapproved exosome products, emphasizing the need for premarket approval similar to drugs. In the EU and China, the use of human‐derived exosomes in cosmetics is prohibited, while South Korea allows them under strict safety standards. Comprehensive safety testing and quality standards are essential to ensure biocompatibility and consistent exosome concentration [23].

Ethical sourcing of exosomes is crucial, requiring proper donor consent and screening for diseases to prevent exploitation. Additionally, regulations on advertising claims are important to ensure that marketing reflects evidence‐based benefits and complies with standards set by agencies like the FDA and EMA [24].

## 7 Future Directions

Future research should prioritize the development and standardization of extracellular vesicle (EV) isolation methods to ensure high purity and yield, refining techniques like differential ultracentrifugation and exploring size‐exclusion chromatography and microfluidics. Standardized protocols will promote consistency across studies.

Enhancing in vivo models is also essential, particularly through the use of larger animal models that better reflect human skin physiology and genetically modified models to understand EV mechanisms. Rigorous clinical trials are necessary to establish the safety, efficacy, and optimal dosing of EV‐based therapies, focusing on long‐term effects and potential adverse reactions.

Collaboration among research institutions, industry, and regulatory bodies will be crucial to advancing these therapies from research to clinical applications, significantly contributing to the field of skin rejuvenation and other medical uses.

## 8 Conclusions

In conclusion, this study underscores the potential significance of blood‐derived exosomes in skin regeneration and rejuvenation. The successful isolation technique using PEG and Dextran, coupled with ultracentrifugation, highlights a promising methodology. Despite advancements, challenges persist in achieving consistent exosome quality and concentration, warranting further research. Comprehensive characterization through NTA, AFM, Cryo‐TEM, and FC provides valuable insights into exosome properties. Positive effects on fibroblast proliferation, collagen, and elastin production in murine and human models support the clinical relevance of blood‐derived exosomes in skin health. Ethical considerations and future perspectives emphasize the need for continued exploration in this evolving field. Overall, this study contributes to advancing our understanding of exosome‐based therapies for skin rejuvenation, but given the small sample size of this pilot study, it would be beneficial to emphasize the importance of future research with larger cohorts to better establish the clinical relevance of the findings.

## Ethics Statement

The study was conducted in accordance with the Declaration of Helsinki and was approved by the Institutional Ethics Committee of N.N. Petrov NMRC of Oncology (10.11.2017, protocol AAAA‐A18‐118012390156‐5). Plasma was obtained from healthy donors in the blood transfusion department.

## Consent

Informed consent was obtained from the subject involved in the study. Written informed consent has been obtained from the patient(s) to publish this paper.

## Conflicts of Interest

The authors declare no conflicts of interest.

## Data Availability

Data are available upon request.
